# Correlation Between Immune Lymphoid Cells and Plasmacytoid Dendritic Cells in Human Colon Cancer

**DOI:** 10.3389/fimmu.2021.601611

**Published:** 2021-02-23

**Authors:** Jing Wu, Hang Cheng, Helei Wang, Guoxia Zang, Lingli Qi, Xinping Lv, Chunyan Liu, Shan Zhu, Mingyou Zhang, Jiuwei Cui, Hideki Ueno, Yong-Jun Liu, Jian Suo, Jingtao Chen

**Affiliations:** ^1^ Institute of Translational Medicine, The First Hospital, Jilin University, Changchun, China; ^2^ Department of Pediatrics, The First Hospital, Jilin University, Changchun, China; ^3^ Department of Stomach Colorectal Anal Surgery, The First Hospital, Jilin University, Changchun, China; ^4^ Department of Pediatric Gastroenterology, The First Hospital, Jilin University, Changchun, China; ^5^ Department of Gynecology, The First Hospital, Jilin University, Changchun, China; ^6^ Department of Cardiovascular Center, The First Hospital, Jilin University, Changchun, China; ^7^ Cancer Center, The First Hospital, Jilin University, Changchun, China; ^8^ Department of Microbiology, Icahn School of Medicine at Mount Sinai, New York, NY, United States; ^9^ Department of Research and Development of Sanofi, Cambridge, MA, United States; ^10^ Key Laboratory of Organ Regeneration & Transplantation of the Ministry of Education, The First Hospital of Jilin University, Changchun, China

**Keywords:** ILC, ILC3, pDC, colon cancer, RNA-Seq, apoptosis

## Abstract

**Background:**

Innate lymphoid cells (ILCs), so far studied mostly in mouse models, are important tissue-resident innate immune cells that play important roles in the colorectal cancer microenvironment and maintain mucosal tissue homeostasis. Plasmacytoid dendritic cells (pDCs) present complexity in various tumor types and are correlated with poor prognosis. pDCs can promote HIV-1–induced group 3 ILC (ILC3) depletion through the CD95 pathway. However, the role of ILC3s in human colon cancer and their correlation with other immune cells, especially pDCs, remain unclear.

**Methods:**

We characterized ILCs and pDCs in the tumor microenvironment of 58 colon cancer patients by flow cytometry and selected three patients for RNA sequencing.

**Results:**

ILC3s were negatively correlated, and pDCs were positively correlated, with cancer pathological stage. There was a negative correlation between the numbers of ILC3s and pDCs in tumor tissues. RNA sequencing confirmed the correlations between ILC3s and pDCs and highlighted the potential function of many ILC- and pDC-associated differentially expressed genes in the regulation of tumor immunity. pDCs can induce apoptosis of ILC3s through the CD95 pathway in the tumor-like microenvironment.

**Conclusions:**

One of the interactions between ILC3s and pDCs is *via* the CD95 pathway, which may help explain the role of ILC3s in colon cancer.

## Introduction

Colon cancer is the third most commonly diagnosed cancer worldwide (1–3); furthermore, its incidence has significantly increased recently (4) and is expected to further rise by 50% in the next five years (5). Epidemiological studies show that the causes of colon cancer are related to environmental, lifestyle, and genetic factors, and that age, intestinal polyps, and ulcerative colitis also represent high-risk factors (6, 7); however, the specific pathogenesis of colon cancer remains unclear.

Currently, treatment of primary colon cancer is mainly surgical; however, postoperatively there is still a risk of recurrence and metastases (8). Therefore, it is important to fully understand the causes of colon cancer to promote the discovery of novel and effective therapeutic targets; this reflects an urgent clinical need from both a theoretical and a practical point of view.

With the rapid rate of discoveries in the field of immunology, cancer immunotherapy has attracted increasing attention (9). The immune system plays an important role in the protection of the host against tumor onset (i.e., tumor immunosurveillance) (10). In addition to tumor cells, stromal and immune cells are also present in the tumor microenvironment, where tumor cells often either recruit or locally induce their proliferation or differentiation to release an array of cytokines that participate in the immune response (10–12). In colon cancer, T and B lymphocytes have been found in proximal colon tumor tissue (5), and natural killer (NK) cells, monocytes/macrophages, dendritic cells (DCs), mast cells, and neutrophils have been detected in the colon tumor microenvironment (12). Notably, the differential distribution of these cells in the tumor microenvironment reflects the diversity of tumor biology (13).

Innate lymphoid cells (ILCs) are a characterized subset of innate lymphocytes (14) that includes three groups: group 1 ILCs (ILC1s) consist of NK cells that express the transcription factor T-bet and secrete interferon (IFN)-γ; group 2 ILCs (ILC2s) express the transcription factor GATA-binding protein 3 and secrete interleukin (IL)-5 and IL-13; and group 3 ILCs (ILC3s) express the transcription factor RAR-related orphan receptor-γt and secrete IL-17 and IL-22 (15–17). ILCs lack an antigen-specific receptor; however, they can still be activated by danger signals from injured mucosal tissue and quickly produce an array of effective cytokines to repel pathogens and tumor cells, thereby sustaining mucosal integrity (18, 19). A previous study has suggested that ILCs may exert both pro- and antitumor functions depending on the phase of cancer and environmental context (20). Until now, most studies on ILCs have focused on mouse models, and very few studies on human colon cancer. Recently, Salimi et al. investigated 13 patients with gastrointestinal (including esophageal, gastric, colon, and rectal) tumors and found a significantly higher frequency of group 1 ILCs (p value: 0.001) in malignant gastrointestinal tumors than in benign tissues (21). Ikeda et al. collected 28 samples from colon cancer patients and reported that the number of NKp44^+^ ILC3s from colorectal cancer tissue was decreased in T3/T4 tumors, with associated decreases in tertiary lymphoid structure induction (22). In this study, we expanded the number of research samples to further study the distribution characteristics of ILCs in colon cancer and their correlation with other immune cells. We investigated the role of ILCs in the colon tumor microenvironment to identify potential strategies for the induction of antitumor immune responses in colon cancer.

## Materials and Methods

### Patient Tissue Specimens

Fresh tumor specimens, including those from tumor-proximal and distal regions, were collected from 58 patients with colon cancer who did not receive radiotherapy or chemotherapy prior to surgery at the Department of Gastric Colorectal Anal Surgery, First Affiliated Hospital, Jilin University (Changchun, China). Patient clinicopathological characteristics were determined according to the National Comprehensive Cancer Network (NCCN) guidelines for colon cancer (Version 2.2018). Proximal tissue was defined as a 2-cm to 5-cm zone bordering the tumor margin. Distal tissue was located >5 cm from the tumor and was considered to be a normal tissue sample and used as a control. There were no restrictions on cancer subtype, age, or sex, and tumor types were identified by histological analysis.

Patients were divided based on TNM stage and tumor histological stage. The TNM staging system is based on the extent of the tumor (T), the extent of spread to the lymph nodes (N), and the presence of metastasis (M). Tumors were classified as stage I, II, III, or IV based on TNM stage and prognosis, where a higher number indicated a more advanced cancer and, likely, a worse outcome. Among them, stage III patients are the most common type. Additionally, the patients were classified as having either glandular or mucous carcinoma.

**Tables 1** and **2** show the clinical characteristics of the patients included in this study. The relationship between these indicators and the frequency of ILCs and plasmacytoid dendritic cells (pDCs) in different patients were analyzed and have been shown in **Tables 1** and **2**.

**Table 1:** Correlations between tumor infiltrating ILC3s and clinicopathological factors of colon cancer.

Factors	N	ILC3s^#^	*P*-value
Age			
< 65	23	0.48 (0.38, 0.75)	0.29
≥ 65	35	0.62 (0.37, 0.98)	
Sex			
Male	32	0.50 (0.29, 0.79)	0.26
Female	26	0.64 (0.41, 0.80)	
Region			
Ascending/Transverse	40	0.62 (0.38, 0.92)	0.34
Descending/Sigmoid	18	0.60 (0.30, 0.74)	
T stage			
T2/T3	37	0.56 (0.40, 0.87)	0.54
T4	21	0.51 (0.33, 0.75)	
N stage			
N0/N1	26	0.51 (0.29, 0.79)	0.38
N3/N4	32	0.67 (0.43, 0.84)	
M stage			
M0	52	0.59 (0.38, 0.87)	0.20
M1	6	0.47 (0.20, 0.65)	
AJCC stage			0.04
I /II	24	0.70 (0.43, 1.27)	
III/IV	34	0.48 (0.31, 0.71)	

^#^The percentage of ILC3s in tumor versus distal tissue.

**Table 2:** Correlations between tumor infiltrating pDCs and clinicopathological factors of colon cancer.

Factors	N	pDCs^#^	*P*-value
Age			
< 65	23	3.14 (1.83, 4.47)	0.08
≥ 65	35	4.49 (2.86, 8.44)	
Sex			
Male	32	4.13 (2.68, 7.87)	0.43
Female	26	3.70 (1.92, 5.44)	
Region			
Ascending/Transverse	40	4.13 (2.32, 5.95)	0.04
Descending/Sigmoid	18	3.15 (2.43, 10.99)	
T stage			
T2/T3	37	4.12 (2.30, 6.55)	0.90
T4	21	3.69 (2.41, 6.39)	
N stage			
N0/N1	26	2.27 (1.44, 4.13)	<0.001
N3/N4	32	4.83 (3.63, 8.07)	
M stage			
M0	52	3.68 (2.26, 5.33)	0.01
M1	6	10.44 (5.37, 14.37)	
AJCC stage			
I /II	24	2.21 (1.30, 3.79)	<0.001
III/IV	34	5.14 (3.69, 8.54)	

^#^The percentage of pDCs in tumor versus distal tissue.

### Tissue Digestion for Single-Cell Suspension

Freshly resected colon tissues from patients with colon cancer and tonsil tissues from children with tonsillar hypertrophy were minced into small pieces in RPMI-1640 medium (Invitrogen, Carlsbad, CA, USA) supplemented with 1% fetal calf serum (Lonza, Basel, Switzerland) and were sequentially digested with collagenase D (1 mg/ml; Sigma-Aldrich, St. Louis, MO, USA) and DNase I (50 μg/ml; Sigma-Aldrich) at 37°C for 40 min and 30 min, respectively. The cell suspensions were then passed through 100-μm and 40-μm cell strainers (BD Biosciences, Franklin Lakes, NJ, USA) to remove debris. The cell suspensions were resuspended in RPMI 1640 medium (Invitrogen) supplemented with 10% fetal calf serum (FCS; Lonza) and 1% penicillin/streptomycin (Sigma-Aldrich) before isolation of mononuclear cells (MNCs) by centrifugation over a Ficoll–Hypaque density gradient centrifugation for 30 minutes at 24°C for further analysis.

### Isolation of ILC3s and pDCs

For further purification, MNCs from freshly resected patient tissue specimens were subjected to Ficoll‐Hypaque gradient centrifugation for 30 min at 24°C. Next, the MNC layer was transferred to a new tube, washed twice with phosphate-buffered saline (PBS), and suspended in PBS. ILCs were sorted using a BD FACSAria system (BD Bioscience) as Lin^−^-enriched MNCs as Lin cocktail^−^ (CD3, CD19, CD20, and CD14), CD94^−^ CD34^−^ CD1a^−^ TCRα/β^−^ TCRγ/δ^−^ CD45^+^ CD127^+^ CRTH2^+/−^ CD117^+/−^ cells using FITC anti-Lin (643510; BD Bioscience), CD94 (305504; Biolegend, San Diego, CA, USA), CD34 (343504; Biolegend), CD1a (300104; Biolegend), T cell receptor (TCR)α/β (306706; Biolegend), TCRγ/δ (331208; Biolegend), allophycocyanin (APC)-H7 anti-CD45 (56017; Biolegend), Percp-cy5.5 anti-CD127 (351322; Biolegend), phycoerythrin (PE)-Cy7 anti-CRTH2 (350118; Biolegend), and BV605 anti-CD117 (562687; Biolegend). pDCs were sorted as Lin^−^ CD94^−^ CD34^−^ CD1a^−^ TCRα/β^−^ TCRγ/δ^−^ CD45^+^ BDCA2^+^ cells using FITC anti-Lin, CD94, CD34, CD1a, TCRα/β, TCRγ/δ, APC-H7 anti-CD45, and APC anti-BDCA2 (17-9818-42; Biolegend). Purity was routinely >99%. Cell viability was determined by trypan blue staining and was >99% after isolation.

### Flow Cytometric Analysis

ILCs and pDCs were identified as described. ILC3s were further divided into NKp44^+^ ILC3s, and NKp44^−^ ILC3s were identified as Lin^−^ CD94^−^ CD34^−^ CD1a^−^ TCRα/β^−^ TCRγ/δ^−^ CD45^+^ CD127^+^ CRTH2^−^ CD117^+^ NKp44^+/−^ cells using AF647 anti-NKp44 (558564; BD Bioscience). Myeloid DCs (mDCs) were identified as Lin^−^ CD45^+^ CD11b^+^ CD11c^+^ cells using FITC anti-Lin, APC-H7 anti-CD45, PE-conjugated anti-CD11b (555388; BD Bioscience), and AF700 anti-CD11c (561352; BD Bioscience). Treg cells were identified as CD45^+^ CD4^+^ CD25^+^ forkhead box (Fox) P3^+^ cells utilizing APC-H7 anti-CD45, PE-Cy7 anti-CD4 (557852; BD Bioscience), FITC anti-CD25 (555431; BD Bioscience), and Percp-cy5.5 anti-FoxP3 (561493; BD Bioscience). B regulatory (Breg) cells were identified as CD45^+^ CD19^+^ CD24^+^ CD38^+^ cells using APC-H7 anti-CD45, APC anti-CD19 (555415; BD Bioscience), BV605 anti-CD24 (562788; BD Bioscience), and PerCP-Cy5.5 anti-CD38 (551400; BD Bioscience). T cells were identified as CD45^+^ CD3^+^ cells utilizing APC-H7 anti-CD45 and PerCP-Cy5.5 anti-CD3 (560835; BD Bioscience). B cells were identified as CD45^+^ CD19^+^ cells using APC-H7 anti-CD45 and APC anti-CD19. NK cells were identified as CD45^+^ CD56^+^ cells using APC-H7 anti-CD45 and AF700 anti-CD56 (557919; BD Bioscience). Monocytes were identified as CD45^+^ CD14^+^ cells utilizing APC-H7 anti-CD45 and FITC anti-CD14 (555397; BD Bioscience). pDCs from tissue and blood were stained with the following monoclonal antibodies: PE-conjugated anti-HLA-DR, and PE-Cy7 anti-CD86 (BD PharMingen, San Jose, CA, USA). Viability was assessed with an Aqua system (BD PharMingen).

For surface marker staining, MNCs were incubated with antibodies at 4°C for 30 minutes and then washed twice before flow cytometric analysis. For the staining of apoptotic marker active caspase-3, cells were stained with a surface marker first and then permeabilised using a Cytofix/Cytoperm kit (BD Bioscience) and stained for intracellular protein caspase-3 with PE-conjugated anti-caspase 3 monoclonal antibodies (BD PharMingen). Fluorescence-associated cell sorting (FACS) plots depict the mean fluorescence intensity values of Ab staining after subtracting the mean fluorescence intensity of the respective isotype control (BD Bioscience).

### Immunohistochemistry

Standard H&E staining was used for colon tissue localisation. Paraffin-embedded, 4-μm-thick tumor sections and tumor-proximal and distal tissue specimens from five patients with colon cancer were selected for immunohistochemistry analysis. Tissue sections were dewaxed and subjected to heat-induced epitope retrieval with preheated antigen-retrieval buffer (pH 9.0; Dako; Agilent Technologies, Santa Clara, CA, USA). Endogenous peroxidase activity was then blocked, and the sections were incubated overnight at 4°C with anti-human BDCA2 (10 μg/ml; clone 124B3.13; Dendritics, Lyon, France). After incubation with a horseradish peroxidase-conjugated secondary antibody (Invitrogen) and development with diaminobenzidine, sections were counterstained with hematoxylin. PBS was used in place of the primary antibody for the negative controls. Images of tissue slides were acquired with a light microscope (BX51N-34-FL-1-D; Olympus, Tokyo, Japan) and analyzed with CellSens Dimension software (Universal Imaging, Bedford Hills, NY, USA).

### RNA-Seq and Analysis

We used freshly sorted ILC3s and pDCs from tumors, proximal and distal regions, and peripheral blood from three patients with stage III colon cancer for RNA-Seq analysis. A total of 200 sorted cells (ILCs or pDCs) were utilized. Cells were sorted into an Eppendorf tube containing 4 μl of lysis buffer (Beijing Genomics Institute, Shenzhen, China) and quickly transferred to liquid nitrogen. RNA-Seq analysis was performed by the Beijing Genomics Institute. The data discussed in this publication have been deposited in NCBI’s Gene Expression Omnibus (23) and are accessible through GEO Series accession number GSE127934 (https://www.ncbi.nlm.nih.gov/geo/query/acc.cgi?acc=GSE127934).

To remove low-quality data, adapters were trimmed using Cutadapt 1 and low-quality bases were removed by ERNE2. To analyse differentially expressed genes, the quality-checked reads were processed using TopHat version 2.0.0 (Bowtie 2 version 2.2.0) as FASTQ files. Reads were mapped to the human reference genome GRCh37/hg19. Read abundance was evaluated and normalized using Cufflinks 3 for each gene, and Cuffdiff from the Cufflinks 2.2.0 package was used to calculate the differential expression levels and to evaluate the statistical significance of these changes in expression. The number of reads per sample is shown in **Tables S1** and **S2**. Only protein-coding genes were considered, and gene level expression values were determined as fragments per kilobase million mapped (FPKM). All genes with FPKM > 1 were designated as expressed and analyzed with an established p-value < 0.05. Pathway enrichment analysis based on the Kyoto Encyclopedia of Genes and Genomes (KEGG) was performed and significantly enriched terms based on low p-values.

### Preparation of Colon Tumor-Derived Supernatant

Single-cell suspensions isolated from three patients with stage III colon cancer were incubated at a final concentration of 2.5×10^6^ cells/ml in complete RPMI in a 6-well tissue culture plate. Tumor supernatant (TS) was collected after 24 hours, filtered at 0.2 µm, and frozen at -80 °C until use.

### Co-Culture of ILC3s and pDCs

ILC3s and pDCs from normal tonsil tissue were prepared and cultured separately at 1×10^6^ cells/ml in complete RPMI (RPMI 1640 containing 10% heat-inactivated fetal bovine serum, 100 U/ml penicillin, 100 mg/ml streptomycin sulfate, 100 U/ml IL-2, and 50 ng/ml IL-7, Cellgro) in the presence or absence of 25% TS, IFN-a (1,000 IU/ml, Millipore), and anti-IFNα (10 ug/ml, Millipore) for 72 hours. Cells and culture supernatant were then harvested for subsequent experiments. Flow cytometric analysis was used for the expression of apoptosis-related genes and the survival rate of ILC3s. ELISA was used to detect the secretion of IL-22 in co-culture supernatant. Giemsa staining was used to detect the morphology of ILC3s and pDCs.

### ELISA

ELISA kits for hIFN-α and hIL-22 (R&D Systems, USA) were used according to the manufacturer’s instructions. For hIFN-α, pDCs were cultured at 2.5×10^6^ cells/ml with TS, TLR7 ligand IMQ (1.5 μM, Invivogen) or anti-IFNα (10 ug/ml, Millipore) in RPMI medium supplemented with 10% FCS, 1% Pen/Strep, nonessential amino acids, sodium pyruvate, and β-mercaptoethanol. Supernatants were collected after 2 days and analyzed with ELISA. For hIL-22, culture supernatant was collected from co-cultured ILC3s and pDCs. All ELISA results are expressed in pg/ml.

### Giemsa Staining

For Giemsa staining, ILC3s and pDCs were seeded on glass coverslips and co-cultured in the presence of 25% TS for 72 hours. Coverslips were air-dried, fixed in methanol, and stained with modified Giemsa stain GS500 (Sigma Diagnostics, USA). Each slide specimen was observed under a light microscope (BX51N-34-FL-1-D, Olympus Corporation, Tokyo, Japan).

### Statistics

Continuous variables were reported as median with interquartile range (IQR), compared using Student’s t-test or Mann-Whitney u-test whenever appropriate. Categorical variables were assessed using the Chi-Square test. Correlation analysis was performed using the Spearman test. All statistical analyses were performed using GraphPad Prism 5 software (GraphPad Software, San Diego, CA, USA). *P* < 0.05 was considered statistically significant.

## Results

### Number of ILC3s in Colon Cancer Tissue Specimens Is Negatively Correlated With Tumor Pathological Stage

**Figure 1:**
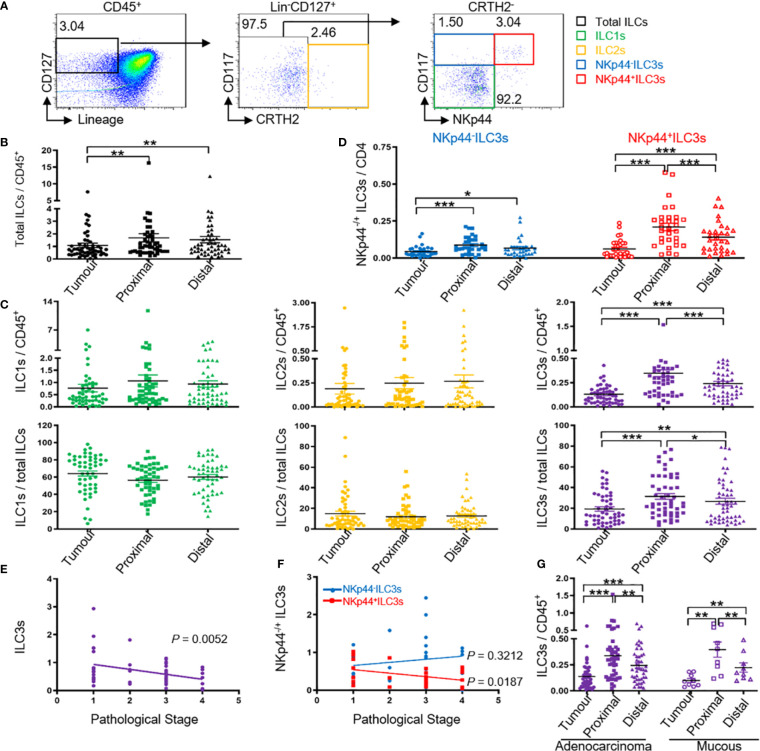
Tumor ILC3s, especially NKp44^+^ ILC3s are negatively correlated with pathological stage. Distribution of ILCs and ILC subtypes by FACS. MNCs from tumor, proximal, and distal regions of 58 patients with colon cancer were prepared. **(A)** The gating used to define ILC subtypes: MNCs were stained for Lin cocktail (CD3, CD14, CD19, and CD20), CD94, CD34, CD1a, TCRα/β, TCRγ/δ, CD45, CD127, CRTH2, and CD117. Total ILCs were identified as Lin^−^ CD94^−^ CD34^−^ CD1a^−^ TCRα/β^−^ TCRγ/δ^−^ CD45^+^ CD127^+^, ILC1s were identified as Lin^−^ CD94^−^ CD34^−^ CD1a^−^ TCRα/β^−^ TCRγ/δ^−^ CD45^+^ CD127^+^ CRTH2^−^ CD117^−^, ILC2s were identified as Lin^−^ CD94^−^ CD34^−^ CD1a^−^ TCRα/β^−^ TCRγ/δ^−^ CD45^+^ CD127^+^ CRTH2^+^, and ILC3s were identified as Lin^−^ CD94^−^ CD34^−^ CD1a^−^ TCRα/β^−^ TCRγ/δ^−^ CD45^+^ CD127^+^ CRTH2^−^ CD117^+^. ILC3s were further divided into NKp44^+/−^ ILC3s. **(B)** ILC levels among the CD45^+^ cells in the indicated tissues. **(C)** Percentage of ILC1s, ILC2s, and ILC3s among CD45^+^ cells and total ILCs in the indicated tissues. **(D)** Percentage of NKp44^+/−^ ILC3s among CD45^+^ cells in the indicated tissues. **(E**, **F)** Correlation between the percentage of ILC3s or NKp44^+/−^ ILC3s in tumor (T) versus distal **(D)** tissue and the pathological stage of cancer. The distal tissue was considered normal tissue and was used for normalization to the background, here T/D. **(G)** Percentage of ILC3s among CD45^+^ cells in colon glandular cancer and mucous carcinoma tissue. In **(B**–**D**, **G)**, each symbol represents the indicated tissue from one patient (circle, tumor; square, proximal region; triangle, distal region). In **(A**, **E**, **F)**, each dot represents one patient. A paired *t*-test and Spearman test were used for statistical comparison. *P < 0.05; **P < 0.01; ***P < 0.001.

In our study, following collection of tissue samples from 58 patients with colon cancer, flow cytometric analysis of MNCs isolated from the tissue showed that nearly 1% of CD45^+^ colon lymphocytes exhibited an ILC phenotype (Lin^−^CD127^+^) (**Figure 1A** and **Figure S1**). Further subtyping revealed that these ILCs comprised chemoattractant receptor-homologous molecules expressed on Th2 cells (CRTH2) ^+^ ILC2s, CRTH2^−^ CD117^+^ NKp44^+/−^ ILC3s, and CRTH2^−^ CD117^−^ NKp44^−^ ILC1s (**Figure 1A**). Additionally, we found that the percentage of total ILCs among the CD45^+^ lymphocytes in the tumor tissue was lower than that in regions proximal and distal to the tumor (**Figure 1B**), with the ILC3 percentage lower than that in the proximal and distal regions (**Figure 1C**) and higher in the proximal region than in the distal region (**Figure 1C**). However, there was no difference in the percentage of ILC1s and ILC2s in the investigated tissue regions (**Figure 1C**). These findings are not consistent with the results of Salimi et al. (21), but consistent with the results of Ikeda et al., which may be due to differences in the investigated tissue samples. Additionally, variations in NKp44^+/−^ ILC3 levels, especially those of NKp44^+^ ILC3s, among the CD45^+^ lymphocytes followed a similar pattern (**Figure 1D**). Because ILC3s can also be classified as CCR6^+/−^, NKp30^+/−^, and NKp46^+/−^ (24), we investigated other subtypes and observed no significant differences in the percentages of the other ILC3 subtypes in the examined regions (**Figure S2A**).

A major prognostic factor for the survival of patients with colon cancer is the pathological tumor stage; therefore, we analyzed possible correlations between ILC3s or NKp44^+/−^ ILC3s and the pathological cancer stage, and observed a negative correlation with ILC3s, especially NKp44^+^ ILC3s, and stage (**Figures 1E, F**; **Table 1**). These data were consistent with previously reported results and showed that natural cytotoxicity receptor (NCR)^+^ ILC3s are more prevalent in stage I/II non-small cell lung cancer than in more advanced-stage tumors, and that they contribute to the formation of protective tumor-associated tertiary lymphoid structures (24). However, in the present study, we found no correlation between NKp44^−^ ILC3s and the pathological stage (**Figure 1F**).

Su et al. (25) reported the clinical significance of circulating immune cells at different colon tumor locations. In the present study, our results showed no correlation between ILC3 percentage and the different tumor regions (**Figure S2B** and **Table 1**). Glandular and mucous carcinomas are common forms of colon cancer. We found similar variations in ILC3 levels among CD45^+^ lymphocytes in colon glandular cancer and mucous carcinoma with the percentage of tumor ILC3s lower than that of proximal and distal ILC3s and the percentage of proximal ILC3s higher than that of distal ILC3s (**Figure 1G**).

### Decreased Numbers of ILC3s in Colon Cancer Tissues Are Correlated With Higher pDC Levels

**Figure 2:**
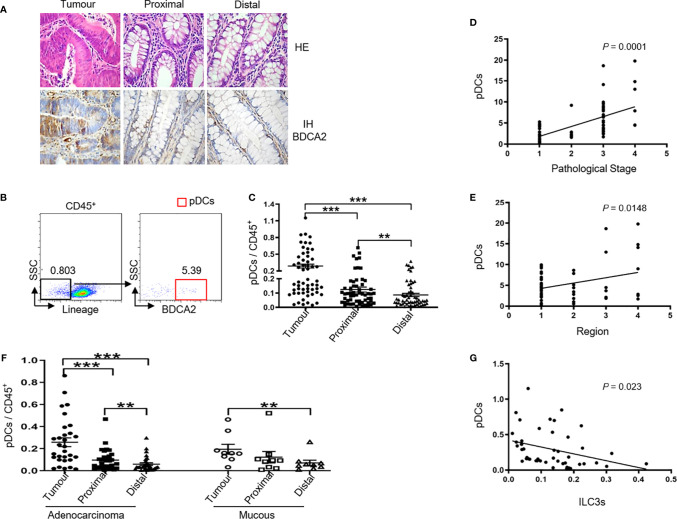
Tumor ILC3s are negatively correlated with tumor pDC levels. MNCs from tumor, proximal, and distal regions of 58 patients with colon cancer were prepared. **(A)** Representative H&E and immunohistochemistry staining of BDCA2 in tumor, proximal, and distal tissues from patients are shown (magnification, 400×). **(B)** The gating used to define pDCs: MNCs were stained for Lin, CD94, CD34, CD1a, TCRα/β, TCRγ/δ, CD45, BDCA2, and pDCs were identified as Lin^−^ CD94^−^ CD34^−^ CD1a^−^ TCRα/β^−^ TCRγ/δ^−^ CD45^+^ BDCA2^+^. **(C)** pDC levels among CD45^+^ cells in the indicated tissues. **(D)** Correlation between the percentage of pDCs in tumor (T) versus distal **(D)** tissue specimens and the pathological stage of colon cancer. **(E)** Correlation between the percentage of pDCs in tumor (T) versus distal **(D)** tissue specimens and the region of the tumor in the colon: 1, 2, 3, and 4 represent ascending, transverse, descending, and sigmoid colon, respectively. **(F)** Percentage of pDCs among CD45^+^ cells in colon glandular cancer and mucous carcinoma tissues. **(G)** Correlation between the number of ILC3s and pDCs among tumor infiltrating CD45^+^ cells. In **(B**, **D**, **E**, **G)**, each dot represents one patient. In **(C**, **F)**, each symbol (circle, tumor; square, proximal region; triangle, distal region) represents the indicated tissue specimen from one patient. A paired *t*-test and Spearman test were used for statistical comparisons. *P < 0.05; **P < 0.01; ***P < 0.001.

pDCs have clinical importance in different tumor types (26) and play a critical role in the tumor microenvironment to promote cancer progression through stimulation of Th2 and regulatory immunity (27). Su et al. (28) showed that the percentage of ILC3s was negatively correlated with pDC levels in lymphoid organs of NRG humanized mice with a persistent HIV-1 infection. Because ILC3 depletion by HIV-1 infection is dependent upon pDCs and IFN-I activity, we hypothesised that the frequency of the two cell types is also correlated in colon cancer. To test this hypothesis, we detected the incidence of pDCs in colon cancer tissues. Flow cytometric analysis of MNCs isolated from colon cancer tissue specimens showed that nearly 0.5% of CD45^+^ colon leukocytes exhibited a pDC phenotype (Lin^−^ CD45^+^ blood DC antigen 2, BDCA2^+^) (**Figure 2B** and **Figure S3**) with the percentage of pDCs higher than that in proximal and distal regions (**Figure 2C**); furthermore, the pDC percentage was higher in the proximal region than in the distal region (**Figure 2C**). Immunohistochemistry staining of BDCA2 revealed that pDCs were present in colon tumor tissue specimens in similar proportions to those obtained by flow cytometry (tumor > proximal > distal; **Figure 2A**).

Additionally, we found a positive correlation between the number of pDCs and pathological tumor stage (**Figure 2D** and **Table 2**). Interestingly, there was also a positive correlation between the percentage of pDCs and the examined tumor region (**Figure 2E** and **Table 2**). Moreover, the variations in pDC levels among CD45^+^ cells in colon glandular cancer tissue and mucous carcinoma were similar, as the percentage of tumor pDCs was higher than that of pDCs in the proximal and distal regions, and the percentage of proximal pDCs was higher than that of distal pDCs (**Figure 2F**).

Next, we determined the correlation between ILC3 frequency and pDCs among tumor infiltrating CD45^+^ cells and observed a negative correlation between the number of ILC3s and pDCs in colon cancer tissues (**Figure 2G**). We then detected the percentage of other immune cells in colon tissues (tumor, proximal, and distal) and found that the percentage of Treg cells among the CD45^+^ cells was higher than that in the proximal and distal regions. However, there was no difference in the percentages of mDCs, CD4^+^ T cells, CD8^+^ T cells, Breg cells, B cells, NK cells, or monocytes among the CD45^+^ cells in the investigated regions (**Figure S4**). Additionally, there was no correlation between the change in the number of ILC3s and that of other immune cells.

### Correlation Between ILC3s and pDCs at the Level of Differentially Expressed Genes (DEGs)

To investigate gene expression in ILC3s, and to further assess the correlation between ILC3s and pDCs in colon cancer tissues, we performed RNA-Seq. The experimental group was tumor-derived (T) ILC3s or pDCs and the control group was distal (D) ILC3s or pDCs.

**Figure 3:**
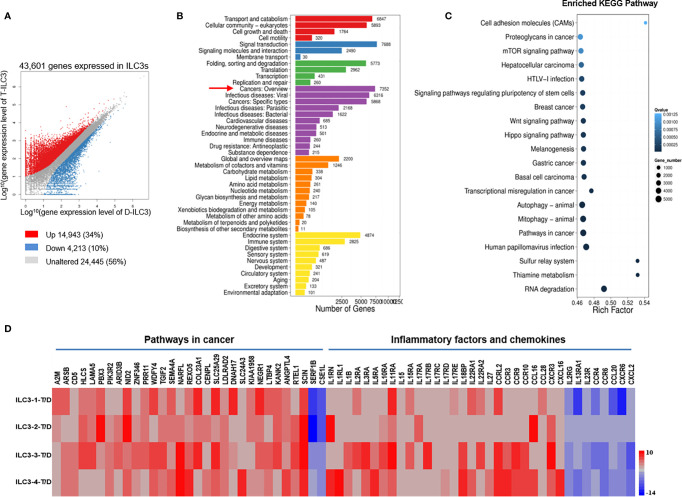
DEGs in ILC3s are associated with both tumor development and inhibition. Analysis of gene expression in tumor and distal ILC3s and identification of significant pathways regulated by the DEGs. **(A)** Scatter plot of DEGs: X and Y axes represent log_10_-transformed gene expression levels. Upregulated, downregulated, and unchanged genes are presented in red, blue, and gray, respectively. **(B)** Pathways regulated by the DEGs: The X-axis represents the number of DEGs, whereas the Y-axis represents the functional classification. **(C)** Pathway functional enrichment of DEGs: The X-axis represents the enrichment factor, and the Y-axis represents the pathway name. The color indicates the q-value (high: white; low: blue). A lower q-value signifies a more significant enrichment. Point size indicates the DEG number. Rich Factor refers to the value of the enrichment factor, which is the quotient of the foreground value (the number of DEGs) and the background value (total number of genes). The larger the value, the more significant the enrichment. **(D)** RNA-Seq analysis of DEGs in tumor versus distal ILC3s. Data from four separate tumor ILC3s (T, experimental group) versus data from four separate distal ILC3s (D, control group).

For the ILC3s, >60 million clean reads were obtained from each sample group with a Q20 score >98% and a mapping rate to the reference genome of each sample varying from 73.89% to 91.40% (**Table S1**), indicating that the data were reliable and could be used for further analysis. A total of 14,943 and 4213 genes were upregulated and downregulated, respectively, in tumor ILC3s relative to distant ILC3s (**Figure 3A**); among them, 7352 genes were related to cancer (**Figure 3B**). Kyoto Encyclopedia of Genes and Genomes (KEGG) pathway analysis confirmed a significant enrichment of genes involved in cancer-associated and RNA-degradation pathways (**Figure 3C**), with more upregulated than downregulated genes. These results show that the tumor environment altered the expression of many ILC3 genes.

After removing genes which could not be confidently mapped to existing entries in any public sequence database, we calculated the log value and fragments per kilobase of transcript per million (FPKM) reads value for each sample. We identified tumor-related genes with significant differences, including 29 upregulated genes and 2 downregulated genes identified from the comparison of tumor and distal regions in four patients (**Figure 3D**). The upregulated genes included those associated with tumor development (*PBX3*, *ARID3B*, *NID2*, *PRR11*, *COL23A1*, *TGIF2*, *SEMA4A*, *COL23A1*, and *SLC25A29*) and inhibition of tumor development (*LTBP4*, *KANK2*, *RTEL1*, *ANGPTL4*, and *SCIN*) (**Figure 3D**), whereas the downregulated genes included one associated with tumor development (*CSE1L*) (**Figure 3D**). These data suggest that ILC3s in tumor tissue might play dual roles during tumor development, in agreement with a previous report (20).

In addition, we analyzed the expression of inflammatory factors and chemokines on ILC3s. The analysis showed that 22 inflammatory genes and 13 chemokines were significantly expressed on ILC3s (**Figure 3D**). Of the inflammatory factors, 19 upregulated genes and three downregulated genes were identified from the comparison of tumor and distal regions in three patients (**Figure 3D**). Of the chemokines, eight upregulated genes and five downregulated genes were identified from the comparison of tumor and distal regions in four patients (**Figure 3D**).

**Figure 4:**
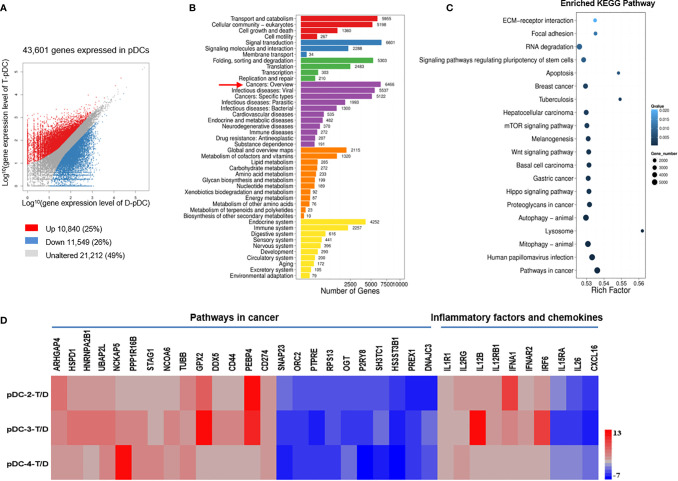
DEGs in pDCs are associated with tumor development. Analysis of gene expression in tumor and distal pDCs and identification of significant pathways regulated by the DEGs. **(A)** Scatter plot of DEGs: X and Y axes represent log_10_-transformed gene expression levels. Upregulated, downregulated, and unchanged genes are presented in red, blue, and gray, respectively. **(B)** Pathways regulated by the DEGs: The X-axis represents the number of DEGs, whereas the Y-axis indicates the functional classification. **(C)** Pathway functional enrichment of DEGs: The X-axis represents the enrichment factor and the Y-axis signifies the pathway name. The color indicates the q-value (high: white; low: blue). A lower q-value represents a more significant enrichment. Point size indicates DEG number. Rich Factor refers to the value of the enrichment factor, which is the quotient of the foreground value (the number of DEGs) and the background value (total number of genes). The larger the value, the more significant the enrichment. **(D)** RNA-Seq analysis of DEGs in tumor versus distal pDCs. Data from three separate tumor pDCs (T, experimental group) versus data from three separate distal pDCs (D, control group). Many of the DEGs in pDCs were associated with tumor development and metastases.

For the pDCs, we obtained >60 million clean reads with a Q20 score >97% and a mapping rate to the reference genome of each sample varying from 75.74% to 88.42% (**Table S2**). A total of 10,840 and 11,549 genes were upregulated and downregulated, respectively, in tumor pDCs as compared with distal pDCs (**Figure 4A**); among them, 6446 genes were related to cancer (**Figure 4B**). KEGG pathway analysis confirmed the enrichment of cancer-associated genes (**Figure 4C**), and as with the ILC3 results, the data show that the tumor environment altered the expression of multiple genes. We identified tumor-related genes with significant differences, including 14 upregulated and 10 downregulated genes from tumor versus distal pDCs in three patients (**Figure 4D**). We found that the upregulated genes were associated with tumor development (*ARHGAP4*, *HSPD1*, *HNRNPA2B1*, *UBAP2L*, *STAG1*, *TUBB*, *GPX2*, *CD44*, *PEBP4*, and *CD274*) (**Figure 4D**), and the downregulated genes were associated with tumor inhibition (*SNAP23*, *PTPRE*, *RPS13*, and *OGT*) (**Figure 4D**). These findings were consistent with previously reported results, showing that pDCs in the tumor microenvironment are associated with the development and maintenance of immunosuppression (27, 29–31).

In addition, we analyzed the expression of inflammatory factors and chemokines on pDCs. Nine inflammatory genes and one chemokine were significantly expressed on pDCs (**Figure 4D**). Of the inflammatory factors, seven upregulated genes and two downregulated genes were identified from the comparison of tumor and distal regions in three patients (**Figure 4D**). Of the chemokines, one downregulated gene was identified from the comparison of tumor and distal regions in three patients (**Figure 4D**).

**Figure 5:**
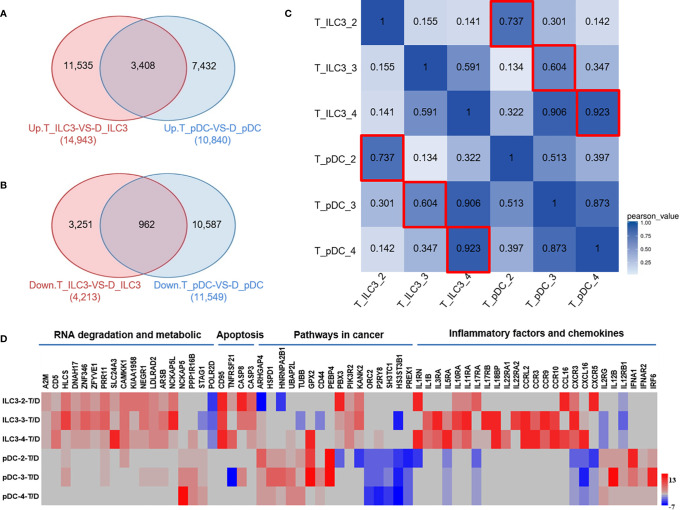
Inverse correlation between ILC3s and pDCs in colon cancer tissues as assessed by RNA-Seq. **(A)** The Venn diagram represents the overlap between the number of upregulated genes in tumor ILC3s versus distal ILC3s (red, left) and tumor pDCs versus distal pDCs (blue, right). **(B)** The Venn diagram represents the overlap between the number of downregulated genes in tumor ILC3s versus distal ILC3s (red, left) and tumor pDCs versus distal pDCs (blue, right). **(C)** Heatmap of Pearson correlations between samples. Both the X and Y axes represent each sample. The colors indicate the degree of the Pearson correlation (high: blue; low: white). **(D)** RNA-Seq analysis of DEGs in tumor ILC3s versus pDCs. Data are from three separate comparisons of tumor ILC3s versus distal ILC3s and tumor pDCs versus distal pDCs.

To assess correlations between ILC3s and pDCs in colon cancer tissues, we further analyzed the RNA-Seq data. Among the upregulated genes, 3,408 genes were co-expressed in ILC3s and pDCs (**Figure 5A**). Among the downregulated genes, 962 genes were co-expressed in ILC3s and pDCs (**Figure 5B**). Moreover, calculation of the Pearson correlation coefficient from RNA-Seq data from all tumor samples revealed an obvious correlation between ILC3s and pDCs in each tissue sample (**Figure 5C**). The analysis of DEGs in ILC3s (**Figure 3C**) versus pDCs (**Figure 4C**) revealed that most upregulated and downregulated ILC3 genes were associated with RNA degradation, metabolic, and apoptotic pathways, whereas most upregulated and downregulated pDC genes were associated with tumor development or inhibition (**Figure 5D**). In addition, some inflammatory factors and chemokines were highly expressed on ILCs or pDCs (**Figure 5D**), particularly ILC3s. These findings were consistent with our flow cytometry results, which showed a negative correlation between the numbers of ILC3s and pDCs in colon cancer tissues (**Figure 2G**).

### pDCs Can Induce Apoptosis of ILC3s in a Tumor-Like Microenvironment

Our results showed a negative correlation between the numbers of ILC3s and pDCs in colon cancer tissues; the number was low for ILCs and high for pDCs (**Figure 2G**). The RNA-Seq results showed that ILC3s showed high expression of apoptosis-related genes such as *CD95*, *TNFRSF21*, *caspase 8*, and *caspase 3*, and pDCs showed high expression of IFN-a-related genes such as *IFNA1*, *IFNAR2*, and *IRF6* in tumors (**Figure 5D**). Su et al. (28) showed that ILC3 depletion by HIV-1 infection is dependent upon pDCs and IFN-I activity. Therefore, we tested whether the low number of ILC3s in the tumor microenvironment is related to pDCs.

**Figure 6:**
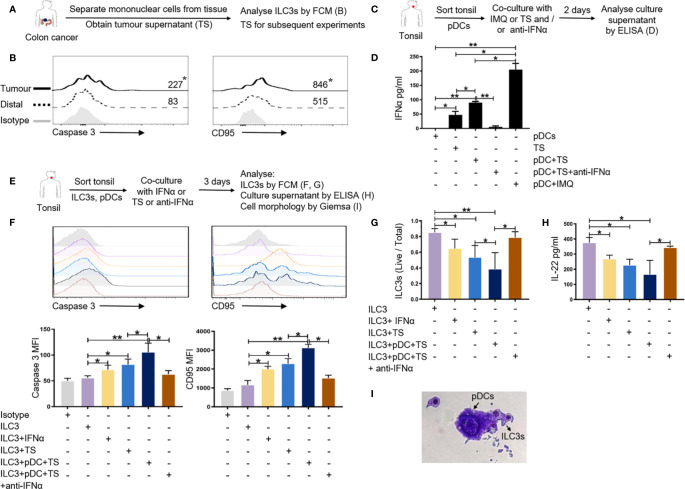
pDCs can affect the survival of ILC3s in the tumor-like microenvironment. **(A, B)** MNCs from tumor and distal regions from three patients with colon cancer were prepared. Flow cytometric analysis was performed for the expression of apoptosis-related genes caspase 3 and CD95 on ILC3s in the tumor and distal tissue control groups. **(C, D)** pDCs from normal tonsil tissue were prepared. TS was added to the pDC culture system and the culture supernatant was collected to detect the secretion of cytokine IFN-a from pDCs by ELISA; IMQ was used as a positive control; anti-IFNα was used to neutralize IFN-a. TS is from tumors of three patients with colon cancer. **(E–I)** ILC3s and pDCs from normal tonsil tissue were prepared. TS, IFN-a, and anti-IFNα was added to the culture system of ILC3s and pDCs. Flow cytometric analysis of the expression of apoptosis-related genes **(F)** and the survival rate of ILC3s **(G)** are shown. ELISA was used to detect the secretion of IL-22 in co-culture supernatant **(H)**. Giemsa staining was used to detect the morphology of ILC3s and pDCs **(I)** (magnification, 50×). Each experiment was repeated three times. A paired *t*-test and Spearman test were used for statistical comparisons. *P < 0.05; **P < 0.01.

First, we used flow cytometry to further verify the expression of apoptosis-related genes in ILC3s in the tumor tissues of colon cancer patients. The results showed that ILC3s overexpressed CD95 and caspase 3 in the tumor tissue compared with that in the distal tumor control group (**Figures 6A, B**). Secretion of cytokineIFN-a was detected in the TS (**Figures 6A, D**).

Previous studies have reported that IFN-a is mainly secreted by pDCs (32). As such, we wanted to detect whether pDCs in the tumor microenvironment secrete IFN-a. Owing to the limited number of pDCs in colon tissue and peripheral blood, we isolated pDCs from normal tonsil tissue for subsequent *in vitro* culture experiments. We added TS to the pDC culture system and used IMQ as a positive control to detect the secretion of cytokine IFN-a. TS was able to promote the secretion of IFN-a from pDCs compared with the TS itself (**Figures 6C, D**). After added anti-IFNα to the pDC culture system with TS, the release of IFN-a is significantly reduced (**Figures 6C, D**).

To test whether pDCs can affect the survival of ILC3s through IFN-a in the tumor microenvironment, we co-cultured pDCs and ILC3s from normal tonsil tissue in the presence or absence of TS, IFN-a, and anti-IFNα to detect the expression of apoptosis-related genes and the survival rate of ILC3s. After co-culturing ILC3s and pDCs with TS or IFN-a, the expression of apoptosis-related genes caspase 3 and CD95 on ILC3s was significantly upregulated (**Figures 6E, F**); the survival rate of ILC3s was significantly reduced (**Figures 6E, G**); and the main factor secreted by ILC3s (IL-22) was also significantly downregulated (**Figures 6E, H**). After added anti-IFNα to neutralize IFN-a, the above effect is obviously weakened (**Figures 6E**–**H**). Giemsa staining results revealed that the ILC3 cell membrane was incomplete and there were scattered apoptotic bodies (**Figure 6I**), this result needs further verification. The above results indicate that pDCs can induce apoptosis of ILC3s through the CD95 pathway by releasing IFN-a in the tumor-like microenvironment.

## Discussion

ILCs are important tissue-resident innate immune cells; the numbers and relative percentages of the three subtypes (ILC1, ILC2, and ILC3) vary in different organs (33, 34). In response to acute environmental challenges and as tissue-resident cells, ILCs can renew and expand in both lymphoid and non-lymphoid organs (34). A change in the ILC population in human tissues is associated with the pathogenesis and progression of chronic infections and inflammatory diseases (18, 28, 35). Recently, Ikeda et al. reported that the number of NKp44^+^ ILC3s from colorectal cancer tissue is associated with tumor-associated tertiary lymphoid structures (22). Our group collected 58 samples from colon cancer patients to further study the distribution characteristics of ILCs in colon cancer and their correlation with other immune cells.

Flow cytometry showed that the numbers of ILC3s and NKp44^+^ ILC3s in colon tumor tissues were lower than those in distal regions and negatively correlated with the pathological stage of cancer; however, there was no correlation between the number of ILC3s and patient age, sex, tumor location, tumor size, lymphatic metastases, or distant metastases. RNA-Seq showed that among the DEGs in tumor versus distal ILC3s, many were associated with tumor development or inhibition (**Figure 3**); however, most of the DEGs were involved in tumor suppression, especially *SCIN*, which was upregulated in tumor ILC3s. These data concur with previous studies suggesting that ILC3s in the tumor microenvironment might have dual functions depending on the cancer phase and environmental context (20, 36–38).

Human ILC3s are the most heterogeneous ILCs. In addition to conventional NK cells, the ILC3 population can also express NCRs and can be divided according to this expression into NKp44^+/−^ ILC3s, NKp30^+/−^ ILC3s, and NKp46^+/−^ ILC3s (24). Additionally, ILC3s can be classified according to the C-C motif chemokine receptor (CCR) 6 expression into CCR6^+^ and CCR6^−^ ILC3s (18). In the present study, changes in the NKp44^+/−^ ILC3 population in tumors and proximal and distal regions were similar to those in total ILC3s, especially the NKp44^+^ ILC3 population; however, changes in the number of ILC1s and ILC2s among the analyzed locations were not significant. These results may be due to insufficient tissue sample size. In future investigations, we will expand the sample size and repeat this analysis.

pDCs are type-I IFN-producing cells that bridge the innate and adaptive immune systems (32) and are specialized in endosomal TLR7/9-mediated recognition of viral nucleic acids with their response involving massive secretion of type-I IFNs to promote virus removal (39). pDCs in the tumor microenvironment mainly exist in a non-activated state and are associated with the development and maintenance of an immunosuppressive environment (27, 29–31). Functional alterations of pDCs in the tumor microenvironment are associated with tumor immune-escape mechanisms (29, 40, 41). In the present study, the number of pDCs in flow cytometric analysis of colon tumor tissues was higher than that in distal regions and positively correlated with tumor location, pathological stage, lymphatic metastases, and especially distant metastases of colon cancer. However, we did not find any correlation between the number of pDCs and patient age, sex, or tumor size. Our RNA-Seq results showed that, among the genes upregulated in tumor pDCs (versus distal pDCs), many were associated with tumor development, whereas many of the downregulated genes were associated with tumor inhibition (**Figure 4**). These data suggest that pDCs might participate in tumor progression and immune escape.

Zhang et al. (28) reported that chronic HIV-1 infection induces ILC3 apoptosis *via* pDC activation, induction of type-I IFN expression, and CD95-mediated apoptosis. Additionally, Maazi et al. (42) showed that pDC activation alleviates airway hyperreactivity and inflammation by suppressing ILC2 function and survival. However, the relevance of ILCs and pDCs in the tumor microenvironment has not been reported. Our flow cytometric data showed a negative correlation between ILC3s and pathological stage and a positive correlation between pDCs and pathological stage. Additionally, we found a negative correlation between percentages of ILC3s and pDCs, with RNA-Seq analysis subsequently confirming this result. The analysis of ILC3 versus pDC DEGs showed that many tumor ILC3 DEGs were involved in RNA degradation, metabolic, and apoptotic pathways, whereas most tumor pDC DEGs were associated with tumor development or inhibition. Julieta et al. (43) reported mRNA degradation as an early apoptotic event in colon cancer, which is concordant with our findings.

In the *in vitro* experiments, after co-culturing ILC3s and pDCs with TS or IFN-a, the expression of apoptosis-related genes caspase 3 and CD95 on ILC3s was significantly upregulated; the survival rate of ILC3s was significantly reduced. In addition to molecules caspase 3 and CD95, other apoptosis-related genes on ILC3s may play important roles in the way pDCs affect ILC3s; this needs further verification. For pDCs, KEGG pathway analysis showed that many of the DEGs were associated with cancer. This supports the results reported by Zhang et al. (28) that pDCs might induce ILC3s apoptosis during chronic HIV-1 infection. Additionally, in the colon cancer environment, pDCs may induce ILC3 apoptosis and promote tumor progression, which would explain the difference in percentage of ILC3s and pDCs in tumor tissues (ILC3s, low; pDCs, high). Moreover, we found multiple upregulated and downregulated genes with similar patterns between ILC3s and pDCs. Pearson correlation analysis of all samples showed obvious correlations between ILC3s and pDCs in colon cancer tissue samples. In our future work, we will confirm these results using *in vivo* experiments.

Su et al. (25) reported that different levels of circulating immune cells are associated with tumor location, stage, differentiation status, and lymphatic metastases in patients with colon cancer. Additionally, they found that the epidemiology, pathogenesis, genetic and epigenetic alterations, molecular pathways, and prognoses differed in patients with left-sided and right-sided colon cancers. In the present study, we found that the percentage of pDCs in the tumor tissue was correlated with the region of the colon with the tumor and that the number of pDCs progressively decreased in the sigmoid, descending, transverse, and ascending colon. However, there was no correlation between the percentage of ILC3s and the region of the colon with the tumor or between the pathological stage and the tumor region (**Figures S2B, C**).

Interestingly, our results showed that the number of ILC3s in the tumor was lower than that in distal and proximal regions, but the number of ILC3s in the proximal region was higher than that in the distal region. Additionally, we found a negative correlation between ILC3s from proximal regions and the pathological stage of cancer (data not shown). RNA-Seq analysis revealed thousands of DEGs between proximal and distal ILC3s (data not shown), including oncogenes. In our future work, we plan to investigate the role of ILC3s in tumor and proximal regions in colon cancer.

## Conclusions

In summary, our data reveal that ILC3s and pDCs represent important cellular components in colon cancer that may participate in tumor progression or inhibition. Specifically, pDCs may induce immune tolerance and promote tumor metastasis in the tumor microenvironment. Furthermore, our findings suggest that ILC3s and pDCs may represent novel therapeutic targets for the modulation of the immune response against colon cancer. Moreover, the identification of ILC3s and pDCs in tumor specimens may represent a new immune score factor to aid in prognostic determination for patients undergoing surgery for colon cancer.

## Data Availability Statement

The datasets presented in this study can be found in online repositories. The names of the repository/repositories and accession number(s) can be found in the article/**Supplementary Material**.

## Ethics Statement

The studies involving human participants were reviewed and approved by Ethical Committee of the First Affiliated Hospital, Jilin University (approval reference 2017-118). The patients/participants provided their written informed consent to participate in this study. Written informed consent was obtained from the individual(s) for the publication of any potentially identifiable images or data included in this article.

## Author Contributions

JTC and JW conceived the study. JW, HC, and GZ performed the experiments and analyzed the data. MZ, HW, JWC, and JS recruited and curated the patient samples and information for flow cytometric. XL and SZ contributed to the flow cytometric studies. JW, CL, and LQ generated and analyzed the RNA-Seq data. JS, Y-JL, and JTC conceived and designed the experiments and supervised the work, with participation from JWC and HU. All authors contributed to data analysis and reviewed and edited the manuscript. All authors contributed to the article and approved the submitted version.

## Funding

This work was supported by the National Natural Science Foundation of China (Grant Nos. 81571534, 81870152, 81901591, and 81800021), the Key Scientific Project of Jilin Province (20140204024YY), the Scientific and Technological Developing Plan of Jilin Province (20160520141JH and 20180101097JC), the 62^nd^ batch of the China Postdoctoral Science Foundation Fund (801171172842), the “13^th^ Five-Year” Science and Technology Research of the Education Department of Jilin Province (YYKH20190043KJ), the Jilin Provincial Key Laboratory of Biotherapy (20170622011JC), the Program for JLU Science and Technology Innovative Research Team (2017TD-08), and the Fundamental Research Funds for the Central Universities.

## Conflict of Interests

The authors declare that the research was conducted in the absence of any commercial or financial relationships that could be construed as a potential conflict of interest.

## Abbreviations

DC, dendric cell; pDCs, plasmacytoid dendritic cells; ILCs, innate lymphoid cells; NK, natural killer; ILC1s, group 1 ILCs; ILC2s, group 2 ILCs; ILC3s, group 3 ILCs; IFN: interferon; IL, interleukin; TCR, T cell receptor; MNCs, mononuclear cells; NCR, natural cytotoxicity receptor; BDCA2, blood DC antigen 2; DEGs, differentially expressed genes; RNA-Seq, RNA sequencing; KEGG, Kyoto Encyclopedia of Genes and Genomes; FPKM, fragments per kilobase of transcript per million; mDC, myeloid DC; Breg, B regulatory; CCR6, C-C motif chemokine receptor 6.
