# Supplementary material for: Correlation Between Immune Lymphoid Cells and Plasmacytoid Dendritic Cells in Human Colon Cancer
Source: Front Immunol. 2021 Feb 23;12:601611. doi: 10.3389/fimmu.2021.601611 (PMC7940519; doi:10.3389/fimmu.2021.601611)
Supplement: Supplementary file 1 [file DataSheet_1.pdf]

Supplementary Figures and Tables

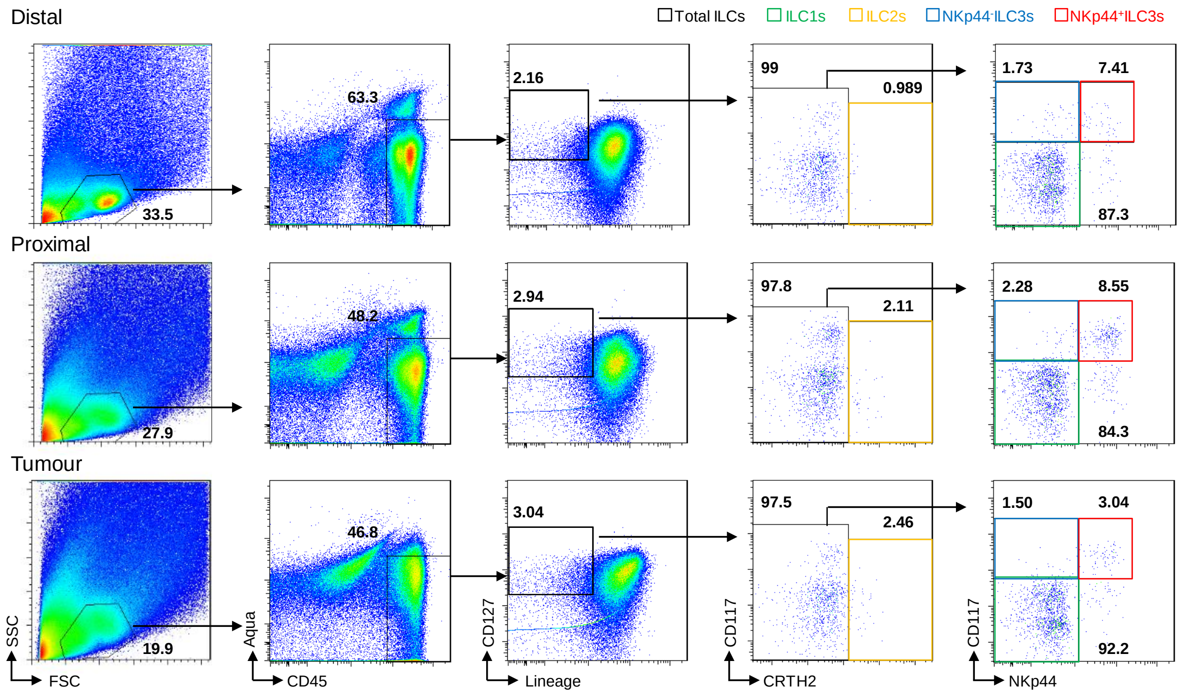

Supplementary Figure 1. Gating ILCs subtypes from tumour, proximal, and distal regions of patients with colon cancer.

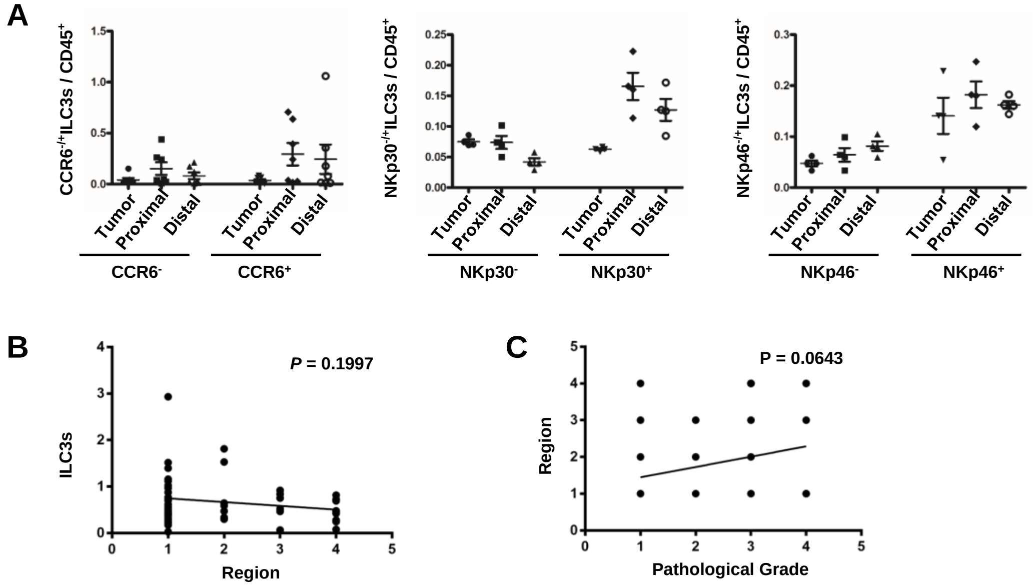

Supplementary Figure 2. (**A**) The percentage of other ILC3 subtypes among CD45^{+} cells in colon cancer tissues, including CCR6$^{+/-}$, NKp30$^{+/-}$, and NKp46$^{+/-}$ ILC3s. (**B**) The correlation between the percentage of ILC3s in tumor (T) versus distal (D) tissue specimens and the region of the tumor in the colon: 1, 2, 3, and 4 represent the ascending, transverse, descending, and sigmoid colon, respectively. (**C**) Correlation between the pathological stage and tumor region in the colon.

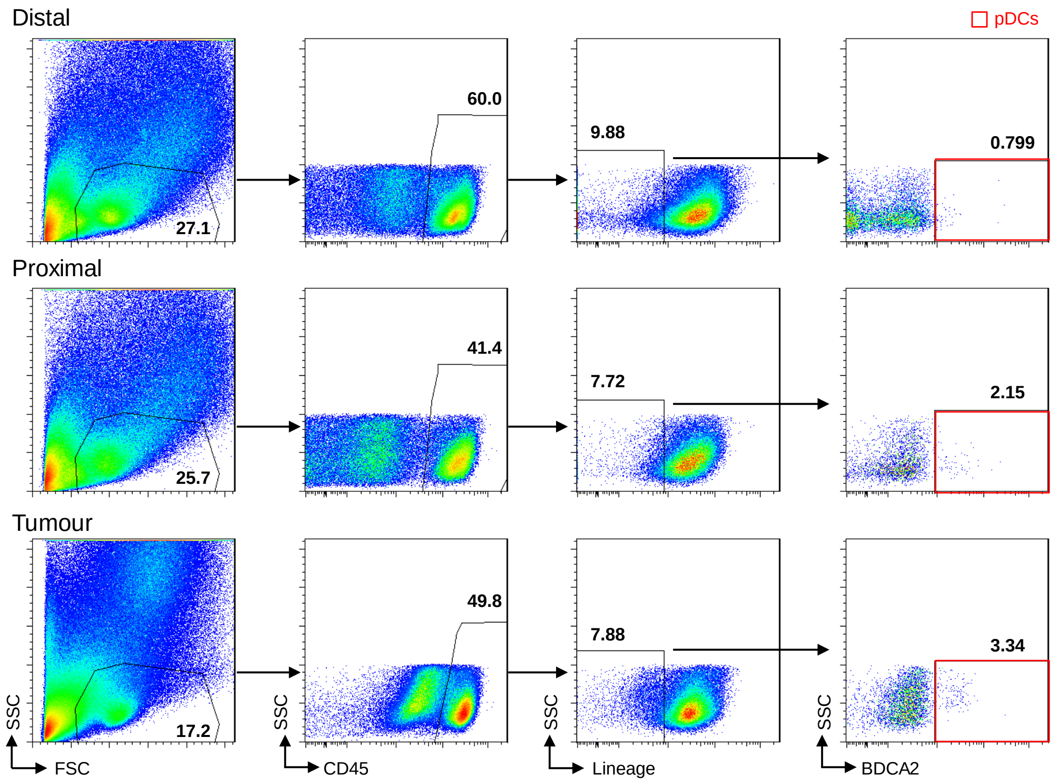

Supplementary Figure 3. Gating pDCs from tumour, proximal, and distal regions of patients with colon cancer.

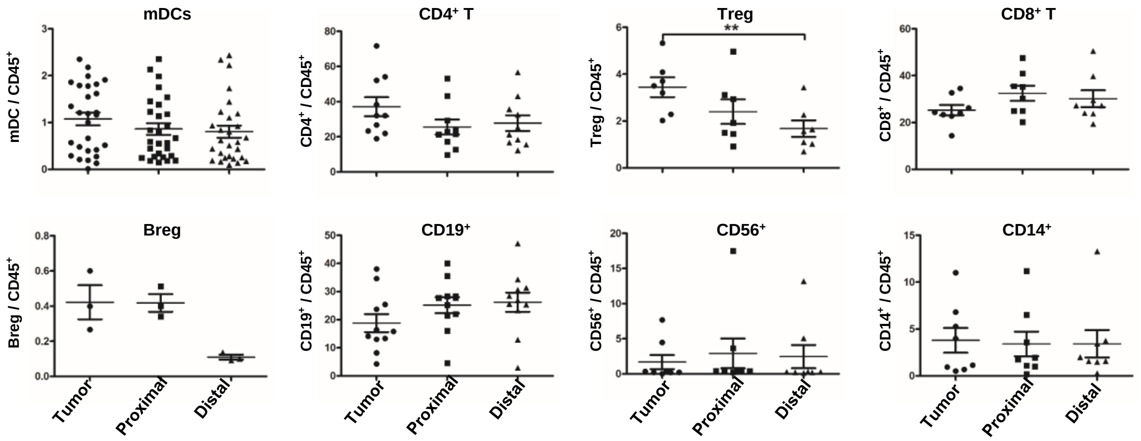

Supplementary Figure 4. Percentage of mDCs, CD4$^+$ T, CD8$^+$ T, Treg, Breg, B, NK, and monocyte cells among CD45$^+$ cells in colon cancer tissues. A paired *t*-test was used for statistical comparisons. $^{**}P < 0.01$.

Table S1. Clean read quality metrics

Sample	Total clean reads (M)	Clean reads Q20 (%)	Clean reads ratio (%)
D_ILC3_1	60.41	99.25	80.03
D_ILC3_2	61.37	98.48	91.40
D_ILC3_3	60.46	98.44	80.50
D_ILC3_4	61.07	99.17	83.12
T_ILC3_1	60.59	99.15	73.89
T_ILC3_2	60.81	98.41	84.36
T_ILC3_3	60.70	99.01	86.46
T_ILC3_4	60.69	99.19	80.81

Table S2. Clean read quality metrics

Sample	Total clean reads (M)	Clean reads Q20 (%)	Clean reads ratio (%)
D_pDC_2	60.84	97.98	88.42
D_pDC_3	61.04	98.23	83.08
D_pDC_4	61.28	99.27	81.59
T_pDC_2	61.18	98.80	79.72
T_pDC_3	60.59	99.08	75.74
T_pDC_4	60.58	98.60	77.30
